# Attribution of weight regain to emotional reasons amongst European adults with overweight and obesity who regained weight following a weight loss attempt

**DOI:** 10.1007/s40519-018-0487-0

**Published:** 2018-02-16

**Authors:** Kirby Sainsbury, Elizabeth H. Evans, Susanne Pedersen, Marta M. Marques, Pedro J. Teixeira, Liisa Lähteenmäki, R. James Stubbs, Berit L. Heitmann, Falko F. Sniehotta

**Affiliations:** 1 Institute of Health and Society, Faculty of Medical Sciences, Newcastle University, Baddiley-Clark Building, Richardson Road, Newcastle Upon Tyne, NE2 4AX UK; 2 MAPP Centre, Aarhus BSS, Aarhus University, Bartholins Alle 10, 8000 Aarhus C, Denmark; 3 Interdisciplinary Centre for the Study of Human Performance (CIPER), Faculty of Human Kinetics, University of Lisbon, Lisbon, Portugal; 4 School of Psychology, Faculty of Medicine and Health, University of Leeds, Leeds, LS2 9JT UK; 5 Research Unit for Dietary Studies at the Parker Institute, Bispebjerg and Frederiksberg Hospital, Copenhagen, Denmark; 6 The Boden Institute of Obesity, Nutrition Exercise and Eating Disorders, University of Sydney, Camperdown, Australia; 7 The Department of General Medicine, Institute of Public Health, University of Copenhagen, Copenhagen, Denmark; 8 Fuse, The UKCRC Centre for Translational Research in Public Health, Newcastle Upon Tyne, UK

**Keywords:** Obesity, Emotion regulation, Weight loss maintenance, Binge eating, Loss of control

## Abstract

**Purpose:**

Despite the wide availability of effective weight loss programmes, maintenance of weight loss remains challenging. Difficulties in emotion regulation are associated with binge eating and may represent one barrier to long-term intervention effectiveness in obesity. The purpose of this study was to determine the relationship between emotion regulation difficulties and the extent of weight regain in a sample of adults who had lost, and then regained, weight, and to examine the characteristics associated with emotional difficulties.

**Methods:**

2000 adults from three European countries (UK, Portugal, and Denmark) completed an online survey assessing self-reported weight loss and regain following their most recent weight loss attempt. They also completed a binge eating disorder screening questionnaire and, if they had regained weight, were asked if they attributed it to any emotional factors (a proxy for emotion regulation difficulties). Spearman’s correlations and logistic regression were used to assess the associations between emotion regulation, weight regain, and strategy use.

**Results:**

Emotion regulation difficulties were associated with greater weight regain (*N* = 1594 who lost and regained weight). Attribution to emotional reasons was associated with younger age, female gender, loss of control and binge eating, lower perceptions of success at maintenance, using more dietary and self-regulatory strategies in weight loss, and fewer dietary strategies in maintenance.

**Conclusions:**

Weight-related emotion regulation difficulties are common amongst regainers and are associated with regaining more weight. Affected individuals are already making frequent use of behavioural strategies during weight loss, but do not apply these consistently beyond active attempts. Simply encouraging the use of more numerous strategies, without concurrently teaching emotion regulation skills, may not be an effective means to improving weight outcomes in this group.

**Level of evidence:**

Level V, descriptive (cross-sectional) study.

## Introduction

Obesity is a major public health concern associated with wide-reaching negative consequences for affected individuals, the health system, and the economy. As of 2015, 68% of men and 58% of women in the UK were classified as overweight or obese, while obesity alone affected roughly a quarter (27%) of the adult UK population [1]. Similar figures for overweight and obesity are found in other western countries, although rates of obesity are lower in most Western European countries than the UK [2, 3]. Elevated weight status confers a range of health risks, including diabetes, cardiovascular disease, and premature death, many of which can be prevented by maintaining a healthy body mass index (BMI) [4]. Weight loss advice and programmes are readily available, and varied approaches can be used to achieve clinically significant weight loss [e.g. 5]. The long-term maintenance of such loss is, however, a harder to achieve target, with only ~ 20% of overweight/obese adults demonstrating success (defined as keeping weight off for at least a year) [6]. To increase effectiveness, weight loss maintenance (WLM) interventions should be grounded in an understanding of the characteristics that are associated with regain, including strategies used in weight loss and WLM, and the perceived barriers to successful WLM.

Emotion regulation is defined as the ability to recognise, understand, and accept emotions; control impulsive behaviours and instead act in line with long-term goals, even in the presence of negative emotions; and to flexibly apply emotion regulation strategies to modulate emotional responses to meet such goals [7]. Differences in emotion regulation are likely to be related to behavioural self-regulation [8] and could help to explain the discrepancy between initially successful weight loss and the subsequent ability to keep weight off. In this context, poor emotion regulation skills may manifest as overeating in response to negative emotions and using food to suppress unpleasant and aversive emotional experiences (i.e. emotional eating) [9], which is one of five ways in which emotions have been found to impact eating behaviour [10]. Emotional eating has been associated with poorer weight outcomes, including higher BMI and lower success in weight loss attempts by means of both diet and surgery [11, 12], while reductions in emotional eating following intervention were associated with greater weight loss [13].

Less research has been conducted regarding the link between emotion regulation or emotional eating and WLM or avoidance of weight regain. In one study, internal disinhibition—which includes both emotional eating and a tendency for dichotomous thinking in relation to eating—predicted weight regain over a year in members of the US National Weight Control Registry (NWCR), all of whom had previously lost > 13 kg and kept it off for at least a year [14]. Emotion-focused coping, including using food to regulate mood and distract from negative emotions, also differentiated obese women who had relapsed from those who maintained their weight loss, in two interview studies [15, 16], and was common among obese women who had undergone, or were awaiting, weight loss surgery following a history of repeated failed attempts at sustained weight loss [17, 18]. Consistent with such evidence, emotion dysregulation has been proposed as one mechanism by which obesity is maintained, as outlined in the clinical obesity maintenance model [19]. Neurophysiological evidence also supports a link between emotion regulation and excess weight [20].

Emotion regulation is linked to a range of psychiatric illnesses, including anorexia nervosa, bulimia nervosa [21], and binge eating disorder (BED) [22], suggesting that many disordered eating behaviours may represent attempts (albeit dysfunctional) at regulating emotions. On the non-clinical end of the spectrum, emotion regulation predicted binge eating behaviours in college students, even after controlling for gender (women were more likely to binge eat) and eating disorder psychopathology (restriction and over-evaluation of weight and shape) [23], suggesting that this association is not confined to diagnosable eating disorders. Despite this, much of the research on the role of emotion regulation in obesity has been limited to samples of treatment-seeking obese individuals with BED [e.g. 22]. In this context, difficulties in generalised emotion regulation (i.e. not specific to eating or weight) were linked to both emotional overeating (i.e. one manifestation of eating-related emotion regulation difficulties) and generalised eating disorder psychopathology [22], although the relationship with weight or weight change following treatment were not assessed. In a review paper, binge eating and mood (both related to emotion regulation) at baseline did not consistently predict weight-related treatment outcomes [24]. The strength of the evidence was weak and follow-up periods were short though, meaning that the impact on long-term outcomes (i.e. maintenance after successful weight loss) was unclear.

While BED is prevalent in overweight and obesity (~ 8%), and higher again in obese individuals seeking weight loss treatment (20–30%) [25], including bariatric surgery [26], subclinical binge eating is also common [25, 27]. That is, overeating that does not involve either the loss of control or objectively large amount of food that is required for a BED diagnosis but may nonetheless be prompted by emotional factors for some individuals. In the NWCR study, internal disinhibition (i.e. emotional eating and dichotomous thinking) predicted regain, even after controlling for the frequency of binge eating, providing some support for the assertion that emotional factors, not just in the form of a BED diagnosis, are important considerations in regain [14]. What remains unclear, however, is whether emotion regulation difficulties and emotional eating also represent a barrier to WLM in a representative population sample of overweight and obese adults, including those who have been less successful at weight loss—that is, individuals whose more modest weight loss (< 13 kg) or failure to keep the weight off for at least a year would initially preclude them from joining the registry. The mechanism via which emotional regulation difficulties may influence regain is also unclear.

Consequently, this study had two main aims: first, to determine the relationship between weight-related emotion regulation difficulties and the extent of weight regain in a general population sample of overweight and obese adults who had successfully lost, and then regained, weight, and whose weight loss had occurred outside the context of a research study (i.e. weight loss RCT) or treatment centre (i.e. commercial weight loss provider, BED treatment, or obesity surgery); and second, to examine the characteristics that were associated with weight-related emotion regulation difficulties in this sample. Here, we were specifically interested in behaviours indicative of BED (e.g. loss of control over eating and eating a large amount of food in a discrete time) but that did not necessarily meet full diagnostic criteria, and the strategies used both in and outside of active weight loss attempts. Based on similar evidence suggesting that emotion regulation difficulties are linked to both high BMI and poorer weight outcomes [11–14, 20], and may be a maintaining factor in obesity [19, 20], it was hypothesised that individuals who demonstrated weight-related emotion regulation difficulties would report greater regain than those without such difficulties. The investigation into the relationship between emotion regulation and strategies for weight loss and WLM was exploratory, due to the absence of prior research to guide hypothesis testing.

The data reported here form part of a wider study, the purpose of which was to describe the self-reported weight trajectories, strategies, and experiences of a group of overweight and obese adults, drawn from the general population, whose recent weight loss attempts had occurred ‘in the wild’ (i.e. not in the context of research, commercial programme, BED treatment, or obesity surgery; the source of most current knowledge about factors relating to weight loss and WLM), and to determine their relationships with weight regain.

## Method

### Participants and procedure

Participants were 2000 individuals aged ≥ 18 years, with a BMI in the preceding 12 months of ≥ 25 kg/m^2^, at least one completed attempt to lose weight in their lifetime, and a weight loss attempt (whether completed or ongoing) in the preceding 12 months. Market research company, Ipsos MORI, was commissioned to build and deliver the researcher-developed online survey, and to recruit and screen nationally representative samples (by age, gender, and geographical location) in each of three participating countries (UK, Denmark, Portugal). Countries were selected based on consortium membership rather than strategically and, as such, cross-country comparisons did not form an explicit study aim.

Existing panel members (i.e. members of the public who had previously and proactively joined the Ipsos MORI participant pool and indicated their willingness to participate in research) in each country were contacted by email and provided with a link to a brief eligibility screening questionnaire. Eligible participants were then directed to the main survey and received points at completion, as per Ipsos MORI standard procedures. Recruitment ceased when the pre-determined study targets for completed surveys had been met (UK: *n* = 1000, Denmark: *n* = 500, and Portugal: *n* = 500). The reason for recruiting a larger sample in UK was to allow for the conduct of analyses to answer secondary, country-specific research questions (to be reported elsewhere). The Newcastle University Faculty of Medical Sciences ethics committee approved the study in the UK (03/09/15; ref: 00902). The University of Lisbon ethics committee approved the study in Portugal (05/11/15; approval number: 45/2015). The Central Denmark Region Committee on Health Research Ethics confirmed that no ethical permissions were required for the study in Denmark. Informed consent was obtained from all participants included in the study prior to accessing the survey.

### Materials

Given that the purpose of the wider study primarily involved the comprehensive description of weight trajectories, strategies, and experiences of the target sample, for the most part, the use of validated questionnaires measuring psychological constructs was deemed unnecessary. Instead, novel items were designed based on previous work with individuals trying to maintain weight loss [28, 29]. Survey respondents completed a purpose-designed online survey including demographic and anthropometric details, weight loss history (age of first weight loss attempt, number of lifetime attempts), a range of self-reported weight variables relevant to their most recent completed attempt (weight prior to previous attempt, lowest weight achieved, weight after regain), strategy use (dietary; e.g. limiting certain types of food, reducing portion size; and self-regulation; e.g. goal setting and self-monitoring of weight and eating) in weight loss and in everyday life, even when not actively trying to lose weight (i.e. akin to maintenance), and perceptions of their success at WLM [30]. Active weight loss and everyday life were differentiated to detect any differences in weight control strategies according to the current intentions of participants, as previous research suggests that success at each task may require a different set of strategies [31].

Respondents who indicated that they had regained weight following their previous weight loss attempt answered an additional question about emotional reasons for regain: “Thinking about why you put weight back on after you’d lost it; which, if any, of the following reasons contributed to your weight regain?” The specific emotional reasons were derived from qualitative work with individuals trying to maintain their weight loss [32] and included: feeling more stressed than usual, feeling more low or down than usual, feeling emotionally drained, feeling frustrated at not being able to lose further weight, turning to food for comfort when experiencing negative emotions, and using food as punishment when experiencing negative emotions. The approach of assessing personal attributions rather than the psychological construct of emotion regulation is consistent with that taken in a study to determine the negative emotional experiences that prompted eating being used as a coping strategy, and therefore, perceived as contributing to further weight gain, prior to bariatric surgery, in a group of obese women [18]. Here, questions were framed specifically in relation to weight regain after an intentional weight loss attempt.

Respondents also completed the BED screening measure from the Patient Health Questionnaire [33], which includes questions on the experience of loss of control over eating and objective overeating (eating an unusually large amount of food in a discrete period of time). It also allows for the categorisation of respondents into ‘at risk’ and non-risk groups, based on meeting the full criteria for a diagnosis of BED. Consistent with a previous study that used this measure [34] and with DSM-5 criteria for BED [35], the frequency criteria for binge eating was relaxed to once per week over the last 3 months (usually twice per week). Importantly, while the PHQ is reliable as a screener, an ‘at risk’ status does not necessarily infer diagnosis but is an indication of the need for further assessment [36]. The survey was developed in English and then forward- and back-translated for administration in Denmark and Portugal.

### Data analysis

For the purposes of this paper, only individuals who reported deliberately and successfully losing and then regaining some weight were included. Weight loss was defined as ≥ 0.45 kg (equivalent to 1 lb which was the smallest amount of weight loss that could be entered in the online survey) that occurred in the context of an attempt of ≥ 1 week duration. The decision to adopt an inclusive position on the definition of successful weight loss was based on the meta-analytic observation that nearly half of all adults in the general population make a deliberate weight loss attempt each year, the prevalence of which increases with increasing overweight and obesity [37], and presumably not all of which will result in large or sustained losses. Further, the maintenance of any weight loss, whether clinically significant (i.e. 5%) or not, is likely to have positive population health effects if achieved at scale [38]. As small losses may reflect the influence of factors other than deliberate weight control practices, the ≥ 1 week duration criteria was added to increase the likelihood that any weight loss did, in fact, result from a completed and intentional effort. Finally, participants whose most recent completed weight loss attempt occurred > 5 years ago were excluded to maximise the accuracy of recall.

Weight-related emotion regulation difficulties (only asked if regain had occurred) were initially operationalised as the total number of emotional reasons that individuals attributed their weight regain to (emotional reasons for regain: ERR; range 0–6), but because this variable was significantly non-normal, it was necessary to dichotomise the data to reflect attribution of regain to at least one ERR vs. no ERR. WLM was operationalised as the change from the lowest weight achieved in the previous weight loss attempt (kg) to the weight after any regain had occurred (unstandardised residualised change score; a higher number indicates a poorer maintenance outcome/more regain). For descriptive purposes, weight after regaining was also expressed as a percentage of the starting weight. Regarding strategies used in weight loss (self-regulatory and dietary) and in WLM (dietary), a score reflecting the total number of each type of strategy endorsed was computed and used in analyses. Details of the specific strategies and the contribution of each to WLM have been described elsewhere [39].

All analyses were conducted in SPSS version 23. The relationship between the total number of ERR and WLM, and with loss of control, binge eating, age, gender, number of lifetime weight loss attempts, and current BMI were assessed using Spearman’s correlations due to the ordinal nature of many of the variables. A logistic regression analysis was then conducted to determine the variables (those from the list above that showed significant univariate relationships with ERR) uniquely associated with attributing weight regain to at least one ERR (vs. no ERR). To determine whether predictive patterns differed for clinically significant weight loss (≥ 5%) versus any weight loss (≥ 0.45 kg), a sensitivity analysis (logistic regression) using only the sample who achieved clinically significant weight loss was also conducted. Although the purpose of the study was not to compare between the three countries, country (dummy coded with Denmark as the reference category, based on differences in attribution to ERR) was controlled for in the regression analyses. Adjusting for multiple comparisons, a significance level of *p* = .01 was set a priori for correlational analyses; *p* = .05 was used to indicate significance in the regression analysis.

## Results

### Descriptive statistics

Of the 2000 survey respondents, based on their self-reported weight loss, 1832 (91.6%) had successfully lost some weight (≥ 0.45 kg) in their previous attempt (≥ 1 week) within the last 5 years, and 1594 (79.7%) of these had regained some weight, and were, therefore, included in the analyses of regain reported here (UK: *n* = 798; Denmark: *n* = 414; Portugal: *n* = 382; 52.4% male; mean age 48.6 years, SD 15.6). The weight characteristics of this sample, by gender, are presented in Table 1.

**Table 1:** Descriptive characteristics of the sample by gender (*N* = 1594)

	Women (*n* = 759)		Men (*n* = 835)	
	Mean (SD)	Range	Mean (SD)	Range
BMI: start of previous attempt	31.7 (6.8)*	18.0–93.1	31.1 (5.4)	21.6–78.4
Weight loss (kg)	9.5 (9.3)	0.5–118.8	9.2 (8.4)	0.5–60.0
Weight loss (%)	10.6 (8.0)***	0.7–53.8	9.0 (6.8)	0.4–42.0
BMI: lowest weight	28.2 (5.9)	15.4–64.5	28.2 (4.6)	18.4–73.5
Regain weight (% of start)	99.2 (9.3)**	53.9–159.6	97.9 (6.2)	68.7–134.0
BMI: after regain	31.2 (6.3)**	19.4–72.6	30.4 (5.3)	22.4–83.3

Gender differences (independent samples *t* tests)

*p < .05; **p < .01; ***p < .001

The mean BMI prior to weight loss and following regain fell in the obese range for both men and women, while the lowest BMI fell in the overweight range. More than a quarter of the sample indicated that they were always trying to lose weight (26.8%), with another 11.2% having tried on 10 or more occasions (1–3 attempts: 30.6%; 4–6 attempts: 26.2%; 7–9 attempts: 3.6%). Women reported a higher number of lifetime attempts than men (*ρ* = 0.198, *p* < .001).

### Emotional reasons for regain, binge eating disorder risk, and strategy use

**Fig. 1:**
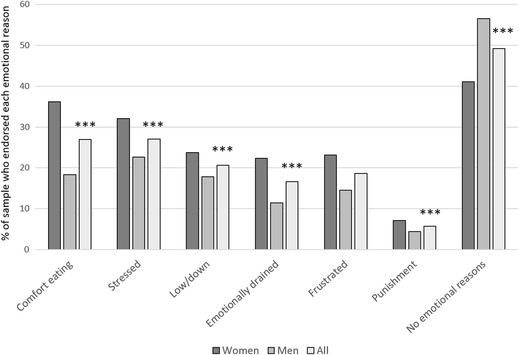
Percentage of the sample who endorsed each emotional reason for regain and relationship with WLM, by gender. ***p < .001 (Spearman’s rank-ordered correlations of relationship between each ERR and weight regain); endorsement of comfort eating, stressed, low/down, emotionally drained, and punishment were each associated with greater weight regain/poorer WLM; no emotional reasons was associated with better WLM/lower regain. The proportion of the sample who endorsed at least one ERR was 56% in the UK, 57% in Portugal, and 35% in Denmark (*χ*^2^ = 59.3, *p* < .001). Significant gender differences were observed for all ERR (*p* = .000–.021)

Half the sample (50.8; 43.5% of men and 58.9% of women) attributed their weight regain to at least one emotional reason (*M* = 1.2, SD = 1.5, range = 0–6). As can be seen in Fig. 1, the most commonly reported ERR was turning to food for comfort when experiencing negative emotions and feeling more stressed than usual, both of which were endorsed by more than a quarter of the sample. Using food as punishment when experiencing negative emotions was endorsed by only a minority of the sample. More than half the sample (54.1%) reported frequently experiencing a loss of control over their eating (criteria 1 for BED), while a quarter (24.5%) reported frequently engaging in binge eating (criteria 2). Only 9.1% of the sample were classified as being at risk of receiving a BED diagnosis, based on also meeting subsequent criteria (i.e. criteria 1 AND 2 at a frequency of at least once a week or more over the past 3 months; AND no engagement in compensatory behaviours).

Participants reported having used a mean of 2.6 self-regulatory strategies (SD = 1.7) and 4.5 dietary strategies (SD = 3.4) as part of their previous weight loss attempt. The mean number of dietary strategies used in WLM (*M* = 3.7, SD = 2.9) was significantly lower than that used in the previous weight loss attempt (*t* = 6.9, *p* < .001).

### Relationships between emotional reasons for regain, self-reported regain, and other variables

As hypothesised, participants who attributed their regain to at least one ERR regained more weight following their most recent weight loss attempt (99.1% of their starting weight following regain, SD = 7.7) than those who did not endorse any ERR (97.9%, SD = 8.0; *t* = − 3.2, *p* = .001), despite no differences in initial weight loss (*p* > .05). As shown in Table 2, the relationship between the total number of ERR (0–6) and regain was also significant. In addition, ERR, higher current BMI, poorer WLM, and female gender were each associated with a higher number of lifetime weight loss attempts (excepting WLM), feeling less successful at WLM, frequent loss of control, frequent binge eating, and being at risk of a BED diagnosis (both excepting gender), as well as the latter three all showing significant relationships with ERR. Older participants were less likely to experience behaviours indicative of BED or to attribute regain to emotional reasons, and tended to use fewer strategies in weight loss but more in WLM, compared to younger participants.

**Table 2:** Spearman’s rank-ordered correlations between ERR and key demographic, weight, and weight loss variables

	ERR	Age	Gender	Current BMI	WLM
Number of ERR (0–6)	–	− 0.25***	0.18***	0.19***	0.13***
Frequent loss of control	0.26***	− 0.21***	0.13***	0.14***	0.09***
Frequent binge eating	0.19***	− 0.19***	0.02	0.14***	0.14***
BED risk status	0.11***	− 0.08**	0.00	0.12***	0.09**
No. lifetime weight loss attempts	0.14***	0.03	0.20***	0.20***	0.05
No. self-regulatory strategies—previous WL attempt	0.16***	− 0.15***	0.12***	− 0.01	0.06
No. dietary strategies—previous WL attempt	0.12***	− 0.10***	0.09***	0.06	0.07**
No. dietary strategies—maintenance	− 0.13***	0.14***	0.01	− 0.09**	− 0.09**
Perceived success at WLM	− 0.19***	0.02	− 0.15***	− 0.27***	− 0.16***

*EER* emotional reasons for regain, *BED* binge eating disorder (0 = no risk; 1 = at risk), *BMI* body mass index, *WL* weight loss, *WLM* weight loss maintenance: unstandardised residualised change score from lowest weight (kg) to regain weight; gender: 1 = male, 2 = female

**p < .01, ***p < .001

More ERR and poorer WLM were both associated with using more dietary strategies in the previous weight loss attempt, and using fewer dietary strategies in WLM. Self-regulatory strategies in the previous attempt were positively associated with ERR but not WLM. Higher current BMI was associated with using fewer dietary strategies in WLM. Participants who endorsed more ERR also reported greater frustration (*ρ* = 0.38, *p* < .001) and lower motivation (*ρ* = − 0.12, *p* = .002) in response to experiencing a temporary lapse during the previous attempt (defined as weight gain of > 1 kg; experienced by 45% of the sample). Greater frustration (*ρ* = 0.11, *p* = .003), but not motivation (*ρ* = − 0.08, *p* = .024), was related to poorer WLM.

### Predicting emotional reasons for regain

The logistic regression to predict ERR (vs. no ERR) was statistically significant (*χ*^2^ = 273.16, *p* < .001, with *df* = 12), indicating that the predictors reliably distinguished between participants who attributed their regain to at least one ERR and those who did not. The model correctly classified 65.9% of cases (67.5% ERR; 64.3% no ERR), increased from 50.8% in the null model. Variables that were associated with greater likelihood of ERR were younger age, female gender, a higher current BMI, frequent loss of control over eating, feeling less successful at maintenance, and the use of a greater number of self-regulatory strategies in the previous weight loss attempt (see Table 3). The strongest psychological predictor was frequent loss of control over eating, with affected participants being 1.8 times more likely to attribute their regain to emotional reasons than those who did not experience loss of control. Consistent with the univariate results, females were more likely than males to attribute regain to emotions (odds ratio =1.4), as were participants from the UK and Portugal compared to those from Denmark (odds ratios of 2.12 and 1.71, respectively). The number of previous weight loss attempts, frequent binge eating, and the number of dietary strategies used in the previous weight loss attempt or in maintenance did not contribute unique variance to the prediction. A sensitivity analysis in the 1126 participants who had achieved clinically significant weight loss (≥ 5%) was significant (*χ*^2^ = 195.96, *p* < .001) and correctly classified a similar proportion of cases (66.7% overall; 67.3% ERR; 66.1% no ERR;). The same pattern of significant predictors was observed.

**Table 3:** Logistic regression analysis predicting attribution to emotional reasons (≥ 1 ERR vs. no ERR)

Variable	*B*	SE	OR (95% CI)	*p*
Age	− 0.02	0.00	0.98 (0.97–0.99)	< .**001**
Gender	0.32	0.11	1.37 (1.10–1.72)	.**005**
Country: UK	0.75	0.14	2.12 (1.62–2.78)	< .**001**
Country: Portugal	0.54	0.17	1.71 (1.23–2.37)	.**001**
Current BMI	0.07	0.01	1.07 (1.04–1.10)	< .**001**
No. lifetime WL attempts^a^	0.06	0.12	1.07 (0.85–1.34)	.591
Loss of control	0.58	0.12	1.79 (1.42–2.26)	< .**001**
Binge eating	0.26	0.14	1.30 (0.99–1.69)	.055
No. self-regulatory strategies	0.09	0.04	1.09 (1.02–1.17)	.**015**
No. dietary strategies: WL	0.00	0.02	1.00 (0.97–1.04)	.874
No. dietary strategies: WLM	− 0.04	0.02	0.96 (0.93–1.00)	.063
Perceived success at WLM	− 0.01	0.00	1.00 (0.99–1.00)	.**023**

Bold values indicate significance at *p* < .05

*ERR* emotional reasons for regain, gender: male = 1, female = 2; *WL* weight loss, *WLM* weight loss maintenance; country (dummy coded): Denmark was chosen as the reference category because fewer participants (35%) endorsed ≥ 1 ERR than those in the UK (56%) or Portugal (57%)

^a^Number of lifetime WL attempts dichotomised: 1–9 vs. 10 or more/always trying

## Discussion

The aims of this study were twofold: to assess the relationship between weight-related emotion regulation difficulties and self-reported weight regain in a general population sample of overweight/obese individuals who had previously lost, and then regained, weight; and to examine the factors that were associated with ERR, to determine the potential mechanisms via which emotion regulation difficulties may impact weight regain. Regarding the first aim and consistent with the hypothesis, participants who attributed their weight regain to at least one emotional reason had regained more weight following their most recent weight loss attempt. There was also a dose–response relationship, whereby a greater number of ERR was associated with greater regain. Additionally, endorsement of most individual ERR (stress, low/down, emotionally drained, comfort eating, and food as punishment, but not feeling frustrated at not being able to lose further weight), as well as reporting greater frustration in response to a temporary lapse, were associated with poorer WLM.

Studies using varied methodologies (qualitative and quantitative) and in non-representative samples (i.e. highly successful weight loss maintainers in the NWCR, and obese individuals with BED or undergoing bariatric surgery) have previously suggested that emotion regulation difficulties play an important role in determining weight outcomes in obesity [13, 17, 19, 20, 22] and are one factor that may differentiate regainers and maintainers following successful weight loss [14–16]. The present results extend this research by demonstrating an association between ERR and greater weight regain in representative samples of adults with overweight and obesity from three European countries who had experienced varying degrees of prior success at weight loss (0.5–119 kg) ‘in the wild’ before regaining at least some of that weight. Consistent with the NWCR study in which internal disinhibition predicted regain after controlling for binge eating frequency [14], ERR, binge eating, and loss of control were all significantly related to weight regain here.

Interestingly, no differences in initial weight loss were observed according to ERR, loss of control, or BED-risk. While it is possible that the regain-specific nature of the ERR questions accounts for this difference with previous research [e.g. 11], it may also be that the time-limited nature of most weight loss attempts allows participants to temporarily override emotional responses to continue behavioural and dietary self-regulation. In contrast, efforts at maintenance should ideally be for life (if regain and the need for further weight loss attempts is to be avoided), meaning that the absence of effective emotion regulation skills may pose a greater threat to sustained weight control.

Regarding the second aim, the analyses assessing the characteristics of individuals who attributed their regain to at least one emotional reason (and who, in turn, had regained more weight) pointed to a cluster of linked but independent risk factors. These included demographic factors such as being younger, being female, and being from the UK or Portugal. Gender differences in emotional eating are well-established [e.g. 17, 40]. Despite endorsing fewer ERR, men were no less likely than women to report frequent binge eating or be at risk for a BED diagnosis. Willingness to report emotional difficulties in men may account for some of this difference [41], or, alternatively suggest that factors other than emotions play a larger role in binge eating in men. It is unclear why differences between countries emerged, although the pattern is consistent with previous findings that Danish females scored lower on a measure of eating disorder symptoms than indicated in US or international norms [42], and lower than Irish participants on an emotional eating scale [43].

Weight- and eating-related factors associated with ERR included having a higher BMI, having a more extensive history of trying to lose weight (often > 10 attempts or always trying), feeling less successful at WLM, and being at risk of a BED diagnosis. Importantly, the experience of frequent loss of control over eating and binge eating were also independently linked to ERR, regardless of whether these were frequent enough to suggest a diagnosable condition. This finding, in combination with the relationship between each of the three variables and weight regain, provides evidence for the relevance of emotion regulation in obesity outside the narrow sample of individuals meeting full diagnostic criteria for BED, on which most of the previous research has been based [e.g. 22]. The associations between a higher number of lifetime attempts, feeling less successful at WLM, more ERR, and greater regain also suggest that reductions in self-efficacy with repeated failed attempts [24] may represent another pathway via which weight loss history and emotions impact behaviour and, in turn, weight outcomes.

Strategy-wise, there are several potential explanations for why individuals with ERR reported using more dietary and self-regulatory strategies during their weight loss attempt, although the cross-sectional nature of the data collection makes it impossible to draw a firm conclusion, and it is likely a complex and bidirectional relationship [8]. First, it may be that, having experienced a loss of control over eating in the presence of a negative emotion, individuals seek to recover from the lapse by increasing their use of self-regulatory and dietary strategies (e.g. by setting new goals and embarking on a new dietary plan) to gain control over their emotions and behaviour. The association between BED-risk status and the number of dietary strategies may also indicate that some participants are engaging in a more extreme restriction-binge eating cycle [10, 44].

Alternatively, from the outset, individuals who are prone to emotional difficulties may take a more chaotic approach to weight loss, whereby many strategies, some of which are probably inconsistent with each other, are used in conjunction to maximise weight loss and/or overcome personally acknowledged emotional difficulties. Rather than recognising the unlikelihood of success based on the unfeasible combination of methods, when regain occurs, this is (at least partially) misattributed to internal factors such as emotions (e.g. feeling stressed or emotionally exhausted, and using food as a comfort) and self-regulatory failure, which prompt further negative emotions. Finally, given that individuals with more ERR were likely to report more lifetime weight loss attempts, including a large proportion who were ‘always trying’, a difficulty in differentiating between attempts may have affected reporting. That is, strategies used across multiple attempts, rather than just the most recent completed attempt, may have been conflated. The observation that a number of lifetime attempts was significantly related to ERR in the univariate but not multivariate analysis, when other variables were accounted for, lends some support to this assertion.

The relationship between ERR and using fewer dietary strategies during maintenance or outside of their active attempts is consistent with an interview study in which regainers exhibited a lack of vigilance over their weight management, a pattern that differentiated them from successful maintainers [16]. Here, it may be that emotional difficulties act as a barrier to continued strategy use in maintenance. Alternatively, the de-regulation of eating behaviour and stopping of many strategies that would support WLM, beyond the active weight loss period (e.g. reducing portion sizes, eating smaller but more frequent meals, and limiting foods high in sugar and fat), is likely primarily responsible for the regain, which is then also misattributed to emotional factors. Together, these findings suggest that one way in which emotions impact weight regain is likely via their effect on the ability to continue behaving in such a way that maintenance of successful weight loss is possible, rather than abandoning behavioural regulation.

### Strengths and limitations

This study had several limitations that should be considered when interpreting the results. First, all weight data were self-reported and there was no opportunity to compare with objective indices. Self-reporting of weight and height are prone to bias [45] and this may, therefore, have over- or under-estimated the rates of successful WLM and affected the associations between BMI, weight regain, and other target variables. To recruit a large representative sample of overweight and obese adults who had attempted weight loss outside of either a treatment (commercial provider, surgery, or BED) or research setting, the options for obtaining weight data were necessarily limited. This survey is the first to assess relationships with emotion regulation in representative general population samples who were purposely not recruited on the basis of their considerable weight loss success, a BED diagnosis, or need for bariatric surgery, and to link the presence of emotional difficulties with the extent of weight regain following weight loss. In contrast, much of the previous research has either been qualitative and the emerging patterns not examined quantitatively in larger samples, or has failed to link the observed emotional difficulties with weight change and specifically regain. Consequently, the associated strengths of this approach go some way to outweighing the potential limitation of self-reported weight data, although replication using objective weights would clearly strengthen the conclusions.

Second, the measure of weight-related emotion regulation difficulties (ERR) was not a validated questionnaire and it is unclear whether it would correspond with measures of more generalised emotion regulation or emotional eating problems. Further, the items assessed emotional attributions for weight regain, which may not reflect the actual reasons for regain—that is, as discussed, there may be a misattribution to emotional factors when, in fact, regain was due to another factor such as the failure to consistently apply dietary and other strategies beyond active weight loss. Nonetheless, understanding people’s attributions for weight regain, regardless of their accuracy, is an important step in matching intervention strategies to expressed support needs, and to increase perceived acceptability and engagement—one of several factors that is likely to determine the success of any weight management approach. Differences in the likelihood of attributing regain to emotions according to non-modifiable factors (e.g. gender and country) also suggest that intervention strategies should be tailored to achieve satisfaction.

Due to the proxy nature of the emotional regulation difficulties measure (attributions), these questions were only asked of participants who self-reported that they had regained weight following their previous weight loss attempt. The analyses used to determine the relationship between ERR and WLM, and the characteristics related to ERR were, therefore, based on a reduced sample, all of whom self-reported that they had regained some weight. It is, however, possible that the participants who had not (yet) regained any weight may also suffer from emotion regulation difficulties that were not captured here. Consequently, the question addressed with the current data is that of whether ERR are associated with the extent of regain amongst regainers, rather than whether emotion regulation difficulties can differentiate regainers from true maintainers (i.e. those who did not regain any weight). The strong associations between regain and frequent loss of control and binge eating, which were assessed in the whole sample, and between these variables and ERR, does, however, imply that the relationship is likely to be generalisable. Finally, the cross-sectional nature of the data means that the direction of associations and question of causality remains unclear, and any interpretations of the data should be taken as tentative and in need of replication using longitudinal or prospective designs and validated/objective measures.

### Implications and conclusions

This representative population study provides further insights into the ways in which emotion regulation difficulties may impact weight regain after weight loss, and the characteristics of individuals who are prone to suffer from such difficulties. The results suggest that emphasis in interventions needs to be placed on addressing the reasons underlying why attempts at continued behavioural self-regulation are failing for some individuals with overweight and obesity, rather than merely encouraging increased use of dietary and self-regulatory strategies without addressing emotion regulation, as this is unlikely to achieve what they have already repeatedly failed to achieve themselves. Interventions should also encourage the personal identification of ways in which different emotions may differentially interfere with weight control, as a simple dose–response relationship (i.e. more emotional reasons, more regain) and blanket teaching of emotion regulation strategies, including recognising and managing distress, for use with all types of emotions is unlikely to tell (or change) the full story.

The recognition of any misattributions of regain to emotional factors, when the primary driver is the behavioural de-regulation that appears common when explicit weight loss intentions are absent, will also be important. Such mastery of emotional experiences so that links with eating and weight are severed, not only for the time-limited period of deliberate weight loss efforts, should allow for the consistent and successful execution of existing behavioural strategies and increase self-efficacy, both of which should have positive impacts on sustained weight control. While the profile of a young female with repeated failed weight loss attempts, low perceptions of success, a high BMI, and a tendency towards loss of control over eating and binge eating, represents a particularly deserving candidate for intervention, the presence of any one of the features associated with ERR will need to be addressed if long-term weight management efforts are to be successful in affected individuals.
